# Recognition of cognitive impairment and depressive symptoms in older patients with heart failure

**DOI:** 10.1007/s12471-020-01527-6

**Published:** 2020-12-15

**Authors:** F. M. M. Oud, P. E. Spies, R. L. Braam, B. C. van Munster

**Affiliations:** 1 Department of Geriatrics, Gelre Hospitals, Apeldoorn & Zutphen, The Netherlands; 2 Department of Internal Medicine, University Medical Centre Groningen, Groningen, The Netherlands; 3 Department of Cardiology, Gelre Hospitals, Apeldoorn & Zutphen, The Netherlands

**Keywords:** Heart failure, Cognitive dysfunction, Dementia, Depression, Depressive symptoms

## Abstract

**Introduction:**

Cognitive impairment and depression in patients with heart failure (HF) are common comorbidities and are associated with increased morbidity, readmissions and mortality. Timely recognition of cognitive impairment and depression is important for providing optimal care. The aim of our study was to determine if these disorders were recognised by clinicians and, secondly, if they were associated with hospital admissions and mortality within 6 months’ follow-up.

**Methods:**

Patients (aged ≥65 years) diagnosed with HF were included from the cardiology outpatient clinic of Gelre Hospitals. Cognitive status was evaluated with the Montreal Cognitive Assessment test (score ≤22). Depressive symptoms were assessed with the Geriatric Depression Scale (score >5). Patient characteristics were collected from electronic patient files. The clinician was blinded to the tests and asked to assess cognitive status and mood.

**Results:**

We included 157 patients. Their median age was 79 years (65–92); 98 (62%) were male. The majority had New York Heart Association functional class II. Cognitive impairment was present in 56 (36%) patients. Depressive symptoms were present in 21 (13%) patients. In 27 of 56 patients (48%) cognitive impairment was not recognised by clinicians. Depressive symptoms were not recognised in 11 of 21 patients (52%). During 6 months’ follow-up 24 (15%) patients were readmitted for HF-related reasons and 18 (11%) patients died. There was no difference in readmission and mortality rate between patients with or without cognitive impairment and patients with or without depressive symptoms.

**Conclusion:**

Cognitive impairment and depressive symptoms were infrequently recognised during outpatient clinic visits.

## What’s new?

- Cognitive impairment and depressive symptoms are highly prevalent in older patients with heart failure.
- Cognitive impairment and depressive symptoms are infrequently recognised during outpatient clinic visits.
- Timely recognition of cognitive impairment and depressive symptoms is important for providing optimal care.

## Introduction

Cognitive impairment and depression are highly prevalent comorbidities in patients with heart failure. A complex interaction exists between heart failure, cognitive impairment and depression. Cognitive impairment and depression in patients with heart failure are known to worsen chronic heart failure and heart failure is known to worsen cognitive impairment and depression. They are also associated with poor outcomes: increased morbidity, readmissions and mortality [1–15].

They share a common pathophysiological basis, such as neurohormonal activation and chronic inflammation and have similar risk factors such as hypertension, diabetes, hypercholesterolaemia and smoking [1–11, 16].

### Recognition and optimal care

Timely recognition of cognitive impairment and depression is important to break the downwards spiral in which patients may find themselves: the treatment regimen of heart failure can be challenging for patients because of the need for self-management, which can be further complicated by cognitive impairment or depression [17]. Poor self-management may lead to exacerbation of heart failure and, as a consequence, more frequent readmissions and even mortality [12, 18, 19]. These exacerbations may in turn lead to an increase in cognitive problems and depressive symptoms. Optimal treatment of depression and support in the case of cognitive impairment could optimise the treatment of heart failure. Conversely, optimal treatment of heart failure may reduce depression and cognitive problems. Consequently, early recognition is the essential first step in improvement of treatment in heart failure patients; it may provide a unique opportunity to improve outcomes for these patients.

### Aim

The aim of our study was to determine if cognitive impairment and depressive symptoms in older patients with heart failure were recognised by their clinicians and, secondly, to establish the association of these disorders with hospital admissions and mortality within 6 months.

## Methods

We performed a cross-sectional cohort study at Gelre Hospitals, a medium-sized community teaching hospital with two locations in the Netherlands. Patients aged ≥65 years diagnosed with heart failure who attended the outpatient cardiology clinic between January 2017 and April 2017 and during a second inclusion period between May 2018 and October 2018 were approached for this study. The gap between these periods was due to logistical problems. At the end of their regular appointment with the cardiologist or nurse specialised in heart failure, patients were asked to participate. Exclusion criteria were communication problems such as severe hearing impairment or speech problems, known severe cognitive impairment, or not being physically well enough to perform or complete the tests. After providing written informed consent, each patient was interviewed by a research student, who was not involved in the patient’s care. This research student administered the Montreal Cognitive Assessment test (MoCA) and the Geriatric Depression Scale questionnaire (GDS). Patient characteristics and cardiovascular risk factors were collected from electronic patient records by a research nurse or research student and through the interviews by a research student. Socio-demographic variables were age, sex and years of education. Cardiovascular risk factors were hypertension, hypercholesterolaemia, current smoking, diabetes mellitus and atrial fibrillation as recorded by a cardiologist in patient records. Severity of heart failure was classified by the cardiologist based on the New York Heart Association (NYHA) guideline. Comorbidities were scored with the Charlson Comorbidity Index [20].

Cognitive status was evaluated with the MoCA [21], a screening instrument for mild cognitive dysfunction that assesses different cognitive domains: attention and concentration, executive functions, memory, language, visuo-constructional skills, conceptual thinking, and orientation. The maximum score is 30 points; a score of 26 or higher is considered normal. We used a cutoff score of ≤22, because we aimed to identify clinically relevant cognitive impairment that may have an impact on a patient’s self-management skills [22, 23].

Depressive symptoms were assessed with the GDS‑2 and GDS-15 [24]. The GDS‑2 is a questionnaire that broadly distinguishes patients with depressive symptoms from patients without symptoms. It is a self-rated screening instrument that consists of two questions regarding feelings of depression and anhedonia in the past month. If a participant answered one of these two questions in the affirmative, the symptoms were further assessed with the GDS-15. This is a 15-item form and a score >5 indicates depressive symptoms.

The cardiologist or nurse was blinded to the test results and was asked to assess whether the patient had cognitive problems or depressive symptoms.

The number of hospital admissions, the number of heart-failure-related admissions and mortality in the 6 months following inclusion were recorded.

The investigation conformed to the principles outlined in the Declaration of Helsinki.

### Analysis

We calculated proportions for categorical variables and means and standard deviations for continuous variables that were normally distributed. For variables that were not normally distributed, we generated medians and interquartile ranges. We compared the characteristics of patients with and without cognitive impairment using the chi-square test, unpaired *t*-test or Mann-Whitney U test where applicable. All analyses were performed using SPSS software version 20.

## Results

**Fig. 1:**
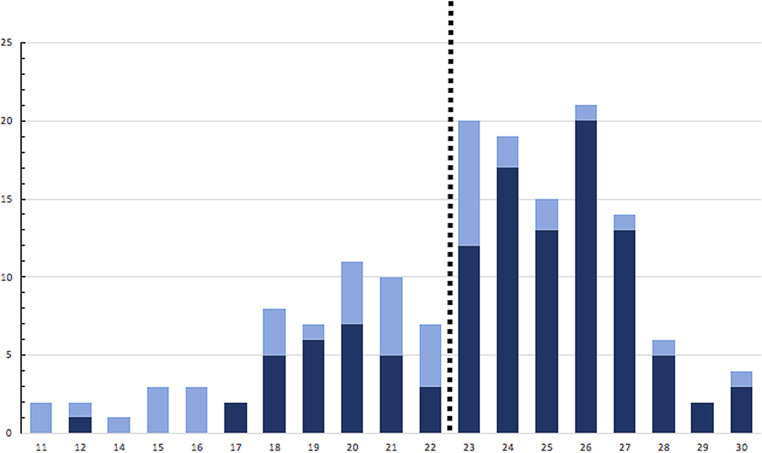
Distribution of Montreal Cognitive Assessment test scores and assessment by clinician. A score of 22 (0–30) or less is indicative of cognitive impairment. Impression of clinician: *dark blue* no cognitive impairment, *light blue* cognitive impairment

A total of 224 patients were asked to participate in our study, of whom 157 agreed. Their baseline characteristics are listed in Tab. 1. Median age was 79 years (range 65–92) and 98 (62%) of participants were male. The majority had NYHA functional class II. The median Charlson Comorbidity Index score was 3 (range 1–9). The median MoCA score was 24, and ranged from 11 to 30 with clustering of the scores around the median (Fig. 1). Cognitive impairment (defined as MoCA score ≤22) was present in 56 of 157 (36%) patients; 109 (69%) scored below the generally used cutoff of 26. In 72 (46%) patients the GDS-15 was performed, after initial assessment by the GDS‑2. The median score on the GDS-15 was 3.5 (range 0–13). Twenty-one patients (13%) had depressive symptoms. Six of these patients had cognitive impairment. During 6 months’ follow-up 44 (28%) patients were readmitted. Twenty-four (15%) patients were readmitted for heart-failure-related reasons and 18 (11%) patients died. There was no difference in overall readmissions and heart-failure-related readmissions between patients with or without cognitive impairment (*p* = 0.62 and *p* = 0.84 respectively) and patients with or without depressive symptoms (*p* = 0.95 and *p* = 0.61respectively). Cognitive impairment and depressive symptoms were not associated with mortality (*p* = 0.83 and *p* = 0.24 respectively).

**Table 1:** Baseline characteristics

	Total *n* = 157	Cognitive impairment *n* = 56	No cognitive impairment *n* = 101	*p*-value
Age, years (median [range])	79 (65–92)	80 (65–92)	77 (65–91)	0.01
Sex (male)	98 (62%)	32 (57%)	66 (65%)	0.31
Years of education (median [range])	10 (5–26)	10 (6–17)	10 (5–26)	0.05
Hypertension	102 (65%)	41 (72%)	61 (60%)	0.11
Hypercholesterolaemia	82 (52%)	35 (63%)	47 (47%)	0.06
Current smoking	49 (31%)	12 (21%)	37 (37%)	0.05
Diabetes mellitus	48 (31%)	20 (36%)	28 (28%)	0.30
Atrial fibrillation	90 (57%)	35 (61%)	55 (55%)	0.21
NYHA class				0.16
I	60 (38%)	16 (29%)	44 (44%)	
II	76 (48%)	33 (59%)	43 (43%)	
III	19 (12%)	7 (12%)	12 (12%)	
IV	2 (1%)	0	2 (2%)	
Comorbidity index (median (range))	3 (1–9)	3 (1–8)	2 (1–9)	0.11
Depressive symptoms	21 (13%)	6 (11%)	15 (15%)	0.47

*NYHA* New York Heart Association

### Recognition of cognitive impairment and depressive symptoms

**Fig. 2:**
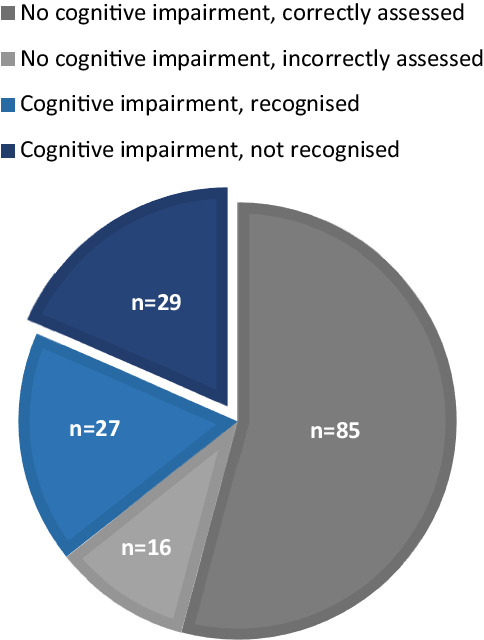
Recognition of cognitive impairment by clinicians. *Grey* patients without cognitive impairment. *Blue *patients with cognitive impairment

Of the 56 patients with cognitive impairment, 29 (52%) were not recognised as such by the clinician during the visit at the outpatient clinic. The sensitivity of assessment of cognitive impairment by the clinician was 48%. The specificity was 84% (Fig. 2).

**Fig. 3:**
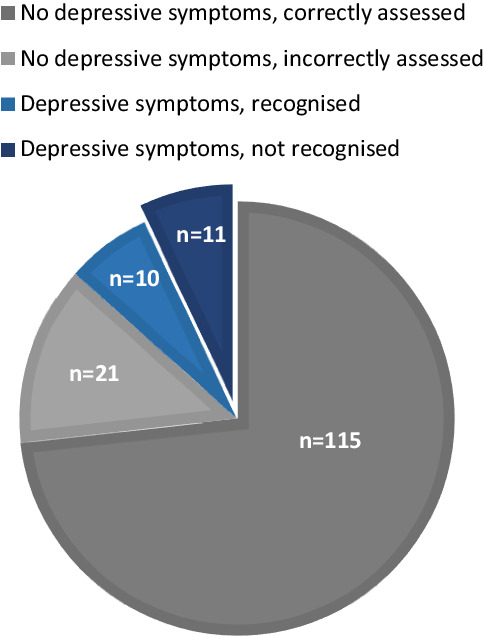
Recognition of depressive symptoms by clinicians. *Grey *patients without depressive symptoms*. Blue* patients with depressive symptoms

Of the 21 patients with symptoms of depression, 11 (52%) were not recognised as such by the clinician. The sensitivity of the assessment of symptoms of depression by the clinician was 48%, the specificity was 85% (Fig. 3).

## Discussion

In this study of older patients with heart failure, we found that cognitive impairment and depressive symptoms were poorly recognised by clinicians during visits to the outpatient clinic. Half of the patients with cognitive impairment and depressive symptoms were not recognised.

Recognition of cognitive impairment and depressive symptoms in patients with heart failure has not received much attention in previous studies. One study in outpatient heart failure patients showed an even poorer recognition of cognitive impairment than our study: cardiologists failed to recognise cognitive impairment in 88% of patients [25]. The difference could be a result of the fact that most of the patients in our study visited the heart failure nurse, who had more time to assess social factors during routine care. Another study, in line with our results, showed that 42% of patients with depressive symptoms were not recognised as such by clinicians in routine care [26].

An explanation for poor recognition of cognitive impairment and depressive symptoms could be that heart failure, cognitive decline and depression share certain symptoms such as loss of energy or decreased physical activities. A cardiologist might attribute these symptoms to heart failure, whereas they could be due to cognitive decline or depression as well [12, 26]. In addition, cardiologists are not routinely trained to diagnose cognitive impairment or affective disorders.

In contrast to our expectations and previous research our study showed no association between cognitive impairment and depressive symptoms and the number of hospital readmissions or mortality during 6 months’ follow-up [12, 18, 19]. This might be due to the relatively small sample size and because previous studies investigated a hospitalised population and had a longer follow-up period.

### Suggestions for clinical practice and for future research

The high prevalence of cognitive problems and depressive symptoms, as well as the poor recognition of these conditions, may lead to suboptimal treatment in a large proportion of older heart failure patients. In addition, suboptimal heart failure treatment can accelerate cognitive decline and worsen depressive symptoms. One of the theories is that, in heart failure patients, low-grade hypoxia, neurohormones and elevated inflammatory cytokines play an important role in development of anatomical brain changes and cognitive dysfunctions [1–15]. Timely recognition may allow adjustment of medication (e.g. statins may not be beneficial in patients with poor prognosis) and lifestyle regimes, simplification of dietary restrictions and better coordination of appointments. In patients with cognitive impairment or depression extra attention should be given to treatment adherence (e.g. medication, fluid restriction). We also recommend that a proxy accompanies the patient to all hospital appointments. These measures may prevent unnecessary hospital admissions and preserve quality of life. Recognition of cognitive impairment is also important in the decision-making process, for example decisions regarding possible implantable cardioverter defibrillator implantation or valve replacement and advance care planning [2, 3, 26–28].

Working towards better recognition starts with awareness and education: cognitive impairment and depressive symptoms are important comorbidities in heart failure. Physicians and nurses should be trained in signalling clinical signs of cognitive impairment and depressive symptoms in patients with heart failure. Barriers to improving recognition and heart failure management could be the physician’s available time, limited geriatric or mental health knowledge, and restricted availability of geriatric or mental health resources [12, 26].

Future research with larger study population sizes and longer follow-up is needed to confirm the current findings. Also, studies are needed to identify the best way to screen for cognitive problems and depression in outpatients with heart failure. As a part of normal care, periodic screening for cognitive decline and depressive symptoms in older patients with heart failure could be introduced in the heart failure care service. Formal cognitive testing, through history-taking by proxy and neuropsychological assessment, should be performed in patients showing symptoms of or screened positive for cognitive impairment. A diagnostic interview should be performed in patients screened positive for depressive symptoms. In all of the suggested strategies a two-step approach between cardiology, geriatric medicine, primary care and mental health care is paramount.

### Study strengths and limitations

The MoCA and GDS are merely screening instruments to assess cognition and mood. We were unable to formally diagnose mild cognitive impairment, dementia or depression in our study. These screening instruments are, however, well suited to screen for problems that indicate a need for further assessment. We deliberately chose a stricter cutoff score of 22 for the MoCA, to make sure that we established the possibility of clinically relevant cognitive impairment that may have an impact on a patient’s self-management skills. Lower cutoffs are supported by previous studies for use in older outpatient populations [22, 23].

Selection bias in this study is possible, as patients with more severe cognitive impairment or depression might be less inclined to participate in the study. This may have led to an underestimation of the prevalence of cognitive impairment and depressive symptoms. However, in a recent meta-analysis the prevalence of cognitive impairment in heart failure cohorts [43% (95% confidence interval 30–55)] was in line with the prevalence in our study (36%) [16]. The prevalence of depressive symptoms in this study (13%) is lower than that described in previous studies (20–42%) [7, 13, 14, 26]. This difference can, in addition to possible selection bias, be explained by a difference in populations. Previous research included only younger patients, patients with higher NYHA class and inpatients. In these populations, a higher prevalence of depressive symptoms is to be expected.

The relatively small study population resulted in low numbers of outcomes during follow-up, which prohibited multivariable regression analyses with adjustment for confounders such as age and comorbidity.

## Conclusion

Cognitive impairment and depressive symptoms in patients with heart failure are frequently present but infrequently recognised by clinicians. The lack of good recognition of these common comorbidities may lead to suboptimal treatment.
